# Phytochemical Profile and Biological Activity of the Ethanolic Extract from the Aerial Part of *Crocus alatavicus* Regel & Semen Growing Wildly in Southern Kazakhstan

**DOI:** 10.3390/molecules27113468

**Published:** 2022-05-27

**Authors:** Zoya Allambergenova, Martyna Kasela, Grzegorz Adamczuk, Ewelina Humeniuk, Magdalena Iwan, Łukasz Świątek, Anastazja Boguszewska, Barbara Rajtar, Aleksandra Józefczyk, Tomasz Baj, Krzysztof Kamil Wojtanowski, Dmitry Korulkin, Kaldanay Kozhanova, Liliya Ibragimova, Zuriyadda Sakipova, Katarzyna Tyśkiewicz, Anna Malm, Krystyna Skalicka-Woźniak

**Affiliations:** 1 School of Pharmacy, S.D. Asfendiyarov Kazakh National Medical University, Almaty 050000, Kazakhstan; zoyaallambergen@mail.ru (Z.A.); kaldanay_k@mail.ru (K.K.); ibragimova.l@kaznmu.kz (L.I.); 2 Department of Pharmaceutical Microbiology, Faculty of Pharmacy, Medical University of Lublin, Chodzki Street 1, 20-093 Lublin, Poland; anna.malm@umlub.pl; 3 Independent Medical Biology Unit, Medical University of Lublin, Jaczewskiego 8b, 20-090 Lublin, Poland; grzegorz.adamczuk@umlub.pl (G.A.); ewelina.humeniuk@umlub.pl (E.H.); 4 Department of Toxicology, Medical University of Lublin, Chodzki 6, 20-093 Lublin, Poland; magda.iwan@umlub.pl; 5 Department of Virology with SARS Laboratory, Medical University of Lublin, Chodzki 1, 20-093 Lublin, Poland; lukasz.swiatek@umlub.pl (Ł.Ś.); anastazjaboguszewska@umlub.pl (A.B.); barbara.rajtar@umlub.pl (B.R.); 6 Department of Pharmacognosy, Faculty of Pharmacy, Medical University of Lublin, Chodzki Street 1, 20-093 Lublin, Poland; ajozefczyk@pharmacognosy.org (A.J.); tbaj@pharmacognosy.org (T.B.); krzysztofkamilw@gmail.com (K.K.W.); 7 Department of Chemistry and Technology of Organic Substances, Natural Compounds and Polymers, Al-Farabi Kazakh National University, 71 al-Farabi Avenue, Almaty 050040, Kazakhstan; dmitriy.korulkin@kaznu.kz; 8 Department of Pharmaceutical Technology, School of Pharmacy, Asfendiyarov Kazakh National Medical University, Tole Be Street 94, Almaty 050000, Kazakhstan; sakipova.z@kaznmu.kz; 9 Supercritical Extraction Research Group, Łukasiewicz Research Network—New Chemical Syntheses Institute, Al. Tysiąclecia Państwa Polskiego 13a, 24-110 Puławy, Poland; katarzyna.tyskiewicz@ins.lukasiewicz.gov.pl; 10 Department of Natural Products Chemistry, Faculty of Pharmacy, Medical University of Lublin, Chodzki Street 1, 20-093 Lublin, Poland; kskalicka@pharmacognosy.org

**Keywords:** *Crocus alatavicus* Regel & Semen, spontaneous flora of Kazakhstan, kaempferol derivatives, antiviral activity, HSV-1, anticancer activity, prostate cancer

## Abstract

The composition of the ethanolic extract from the aerial parts of *Crocus alatavicus* Regel & Semen from southern Kazakhstan spontaneous flora was analyzed together with the determination of its antibacterial, antifungal, antiviral and anticancer activity. The phytochemical profile analysis by high-performance liquid chromatography-electrospray ionization-quadrupole-time of flight-mass spectrometry (HPLC/ESI-QTOF-MS) revealed the presence of multiple kaempferol derivatives. High-performance reverse-phase liquid chromatography combined with a photodiode-array detection (RP-HPLC/PDA) found that kaempferol 3-*O*-dihexoside and kaempferol 3-*O*-acyltetrahexoside accounted for 70.5% of the kaempferol derivatives. The minimum inhibitory concentration (MIC) values of the extract for all the tested reference microorganisms were high, reaching 10 mg/mL for yeasts and 20 mg/mL for bacteria. In contrast, antiviral activity was observed at 2 mg/mL, resulting in the inhibition of the HSV-1-induced cytopathic effect and the reduction in virus infectious titer by 1.96 log, as well as the viral load by 0.85 log. Among the tested prostate cancer cell lines, significant cytotoxic activity of the extract was noted only on the LNCaP cell line, with an IC_50_ value of 1.95 mg/mL. The LNCaP cell line treated with 2 mg/mL of the extract showed a noticeably reduced number of spindle-shaped cells with longer cellular projections, a significant increase in the peak corresponding to the population of apoptotic cells in the sub-G1 phase and a decreased intracellular glutathione (GSH) level, suggesting the prooxidative properties of the extract. The obtained data provide novel information about the flavonoids present in the aerial part of *C*. *alatavicus* and suggest its potential application as a source of the compounds active against HSV-1 and metastatic, androgen-sensitive prostate cancer.

## 1. Introduction

The genus *Crocus,* which belongs to the *Iridaceae* family, comprises about 85 species that occur in Europe, the Middle East, and North Africa. Among the *Crocus* species, the most extensively studied is *C*. *sativus* L., commonly known as saffron, with well-documented phytochemical and pharmacological data. This crocus is widely cultivated in several countries, especially Southern Europe and Central Asia [1,2]. *C*. *sativus* and its main constituents, such as safranal, crocins, and crocetin, could be considered an effective treatment against various diseases, including civilization disorders [1,3,4,5,6].

However, there are only limited data on *Crocus alatavicus* Regel & Semen concerning its phytochemistry and biological activity [2,7,8]. It should be noted that this endemic and rare crocus, known as alatau or white saffron, is an early spring ephemeral and geophytic-geocarpic plant that grows in mountain meadows in several central Asian countries, including Kazakhstan [9].

The aim of the present paper was to investigate the antimicrobial, antioxidant and anticancer activity of the ethanolic extract obtained from the aerial parts of *C*. *alatavicus* growing wildly in southern Kazakhstan. For the first time, the obtained results were analyzed in the aspect of the phytochemical data concerning the composition and quantity of flavonoids.

## 2. Results

### 2.1. Phytochemical Analysis

**Figure 1:**
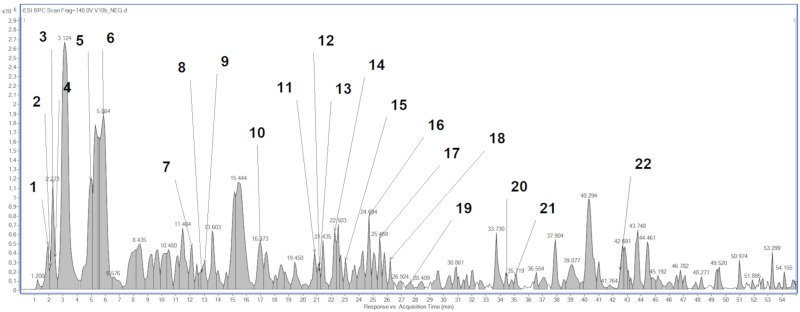
Base peak chromatogram of *Crocus alatavicus* (CA) extract by high-performance liquid chromatography-electrospray ionization-quadrupole-time of flight-mass spectrometry (HPLC/ESI-QTOF-MS).

Phytochemical profile analysis by high-performance liquid chromatography-electrospray ionization-quadrupole-time of flight-mass spectrometry (HPLC/ESI-QTOF-MS) revealed the presence of 22 compounds (Figure 1, Table 1). Except multiple kaempferol derivatives, such as kaempferol 3-*O*-dihexoside and kaempferol 3-*O*-acyltetrahexoside, the CA extract consisted of other compounds, such as flavonoids (e.g., rutoside), organic acids (e.g., citric acid) or monoterpenoids (crocusatin).

The flavonoid content in the ethanolic extract from the aerial parts of *Crocus alatavicus* is presented in detail in Table 2. High-performance reverse-phase liquid chromatography combined with a photodiode-array detection (RP-HPLC/PDA) revealed that kaempferol derivatives accounted for as much as 96.5% of all the identified flavonoid compounds. Moreover, kaempferol 3-*O*-dihexoside and kaempferol 3-*O*-acyltetrahexoside accounted for 70.5% of all the identified kaempferol derivatives.

### 2.2. Antibacterial and Antifungal Activity

The activity of CA extract against Gram-positive (*Staphylococcus aureus*, *S. epidermidis*, *Bacillus cereus*, *Cutibacterium acnes*) and Gram-negative (*Escherichia coli*) reference bacteria, as well two reference species of yeasts (*Candida albicans* and *C. glabrata*), was studied. The minimal inhibitory concentration (MIC) values for all the tested reference microorganisms were comparable and ranged from 10 mg/mL for yeasts to 20 mg/mL for bacteria (Table 3). There was no difference between MIC and minimal bactericidal concentration (MBC) values between methicillin-susceptible *S. aureus* ATCC 29213 and methicillin-resistant *S. aureus* ATCC BAA-1707. For all the tested bacteria, except *E. coli* and *S. epidermidis*, the MBC and MIC values were equal, which means that the tested compound exhibited bactericidal properties. The analogous situation was found for reference yeasts *C. albicans* and *C. glabrata*, where minimal fungicidal concentration (MFC) to MIC ratios ranged from 1 to 2, indicating fungicidal activity.

### 2.3. Antiviral Activity

The CA extract exerted low cytotoxicity towards VERO cells, both after 24 and 72 h of incubation, with comparable CC_50_ values (50% cytotoxic concentration) of 2.49 ± 0.05 and 2.48 ± 0.01 mg/mL, respectively.

**Figure 2:**
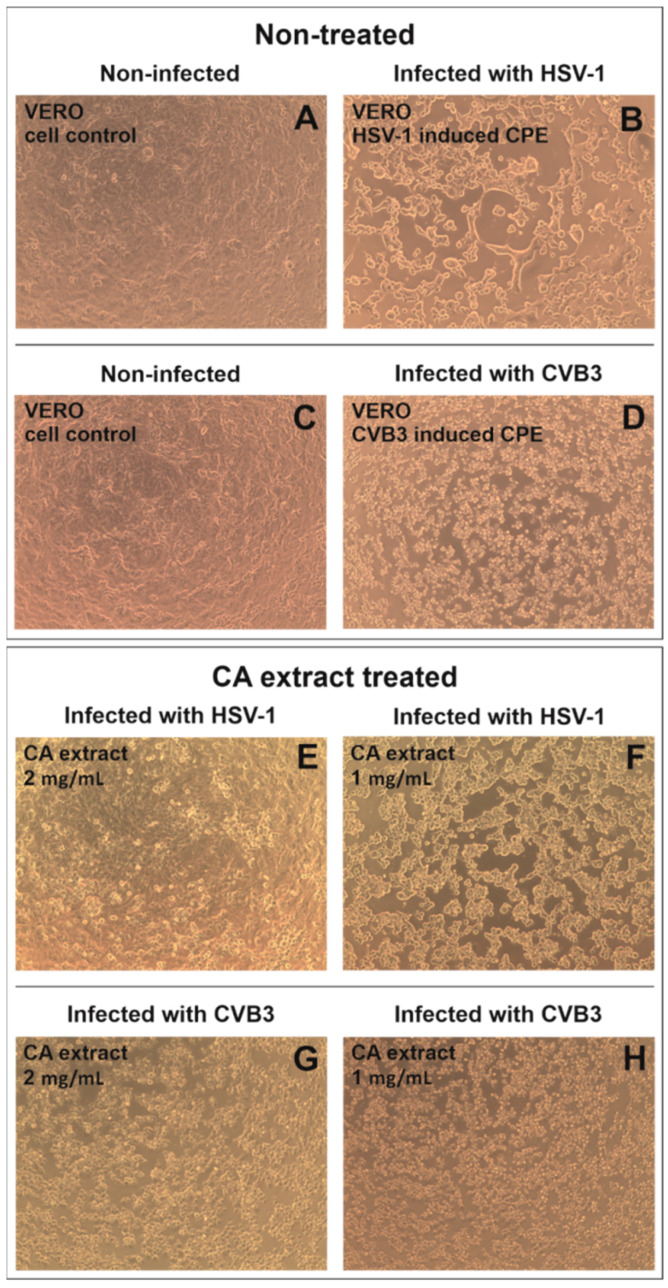
The influence of CA extract on HSV-1 or CVB3-induced cytopathic effect (CPE) formation in infected VERO cells; (**A**,**C**) non-infected VERO cells, (**B**) HSV-1 induced CPE in VERO cells, (**D**) CVB3 induced CPE in VERO cells, the influence of CA extract 2 mg/mL (**E**) and 1 mg/mL (**F**) on HSV-1 induced CPE in VERO cells, the influence of CA extract 2 mg/mL (**G**) and 1 mg/mL (**H**) on CVB3 induced CPE in VERO cells. (Magnification × 100).

During the antiviral studies, the VERO cells were infected with herpes simplex virus 1 (HSV-1) or coxsackievirus B3 (CVB3) and treated with the CA extract in the highest non-toxic concentration of 2 mg/mL and also twofold dilution—1 mg/mL. As can be concluded from Figure 2, the CA extract did not show any significant effect on the occurrence of the CVB3-induced cytopathic effect (CPE) in infected VERO cells, and only at 2 mg/mL, the cells appear to be less rounded (Figure 2G) than in the virus control (Figure 2D). However, the concentration of 2 mg/mL of the tested extract visibly inhibited (Figure 2E) the occurrence of HSV-1 incited CPE in comparison with the HSV-1 virus control (Figure 2B), and the SI (selectivity index) was calculated to be 1.24. The antiviral effect appeared to be dose-dependent, and at 1 mg/mL, no CPE inhibition was observed (Figure 2F).

**Figure 3:**
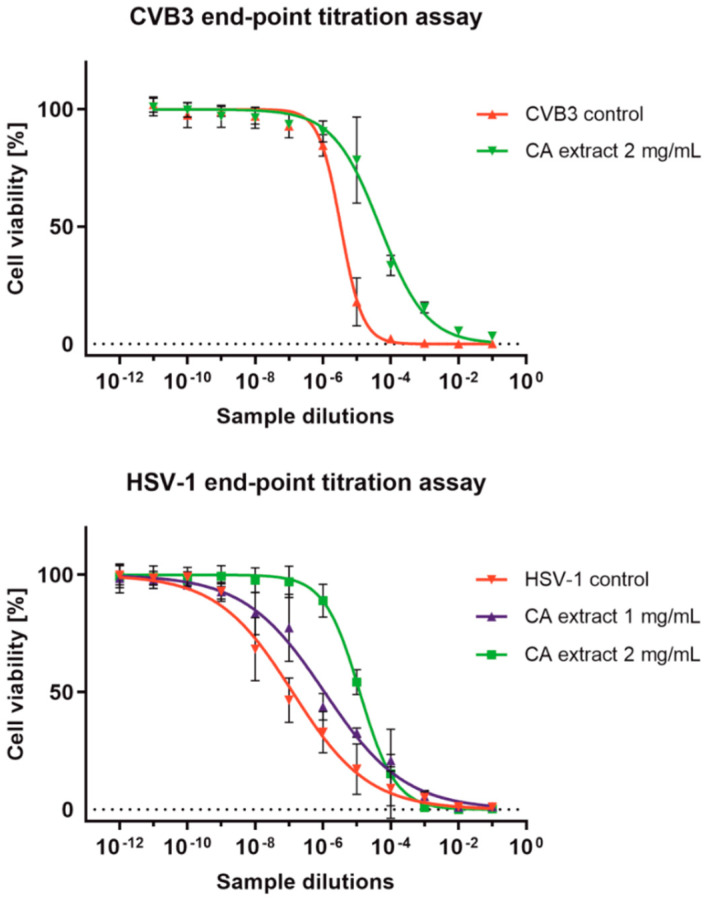
Endpoint dilution assays of CVB3 and HSV-1 titers in samples treated with CA extract.

Subsequently, end-point dilution assays of CVB3 and HSV-1 titers in samples treated with CA extract were performed (Figure 3). The extract at 2 mg/mL decreased the infectious titer of CVB3 by 1.14 log (log CCID_50_/mL) in comparison to the virus control. However, the same concentration resulted in the reduction in HSV-1 infectious titer by 1.96 log when compared to the virus control (Table 4). The replication of both viruses was dose-dependently inhibited by the CA extract; however, significant antiviral activity can only be reported for the samples that inhibit the infectious titer by at least 3 log.

### 2.4. Anticancer Activity

#### 2.4.1. Cytotoxic Evaluation

**Figure 4:**
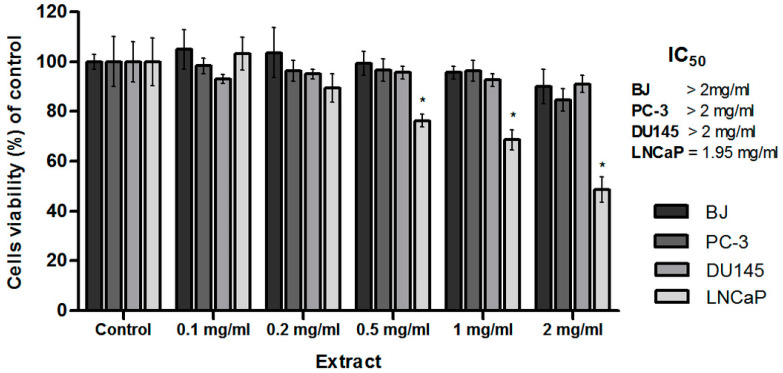
Skin fibroblast cell line (BJ) and three prostate cancer cell lines (PC-3, DU145 and LNCaP) viability (% of control) based on the MTT assay treated with CA extract. The cells were treated with an extract in a wide range of concentrations (2–0.1 mg/mL) or DMSO as a vehicle in control cultures for 48 h. The values were presented as mean ± SD derived from three independent experiments. * *p* ≤ 0.05.

The 3-(4,5-dimethylthiazol-2-yl)-2,5-diphenyltetrazolium bromide (MTT) test was conducted to assess the cytotoxicity of the CA extract on three prostate cancer cell lines (PC-3, DU145, LNCaP) and skin fibroblast cell line (BJ) after 48 h of incubation. All the tested cell lines were treated with CA extract in a wide concentration range of 0.1–2 mg/mL (Figure 4). Skin fibroblast cells were used to evaluate the cytotoxicity of the tested extract towards normal cells. The CA extract did not cause a statistically significant decrease in the viability of BJ cells, even in the highest concentration—2 mg/mL.

Among the tested prostate cancer cell lines, significant cytotoxic activity of the extract was noted only on the LNCaP cell line. In addition, the viability of the LNCaP cells decreased in a dose-dependent manner. The half-maximal inhibitory concentration (IC_50_) value of the tested extract determined with the AAT Bioquest IC50 calculator for the LNCaP cell line was 1.95 mg/mL. In the case of the PC-3 and DU145 prostate cancer cell lines, the IC_50_ value was not achieved. Therefore, the CA extract in the concentration of 2 mg/mL on LNCaP cells was selected for further studies.

#### 2.4.2. Assessment of Cell Morphology

**Figure 5:**
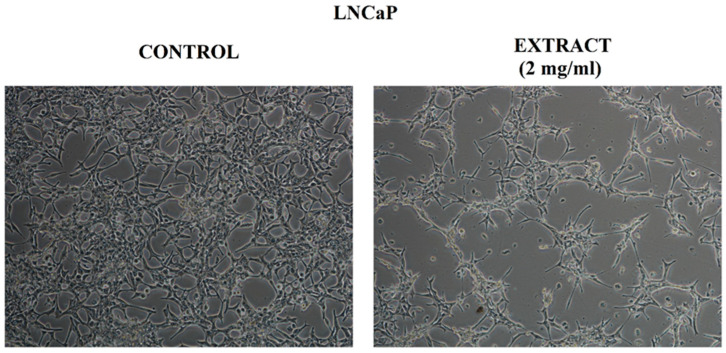
LNCaP cell morphology treated with CA extract or DMSO as a vehicle in control cultures for 48 h. They were analyzed using a phase-contrast microscope, Nikon Eclipse Ti. (Magnification × 100).

The microscopic observation of the tested LNCaP cells treated with the extract at a concentration of 2 mg/mL confirmed the results obtained in the MTT test. The vehicle-treated control LNCaP cells revealed epithelial-like morphology (Figure 5). The cells adhered closely to the surface of the culture plate and to each other. The microscopic image of the LNCaP cells treated with the extract showed a noticeably reduced number of cells compared to the control cells. In the case of the morphological changes in cells, a more spindle-shaped shape and longer cellular projections were visible. In the field of view, some round and floated cells in the medium were observed.

#### 2.4.3. Cell Cycle Analysis

**Figure 6:**
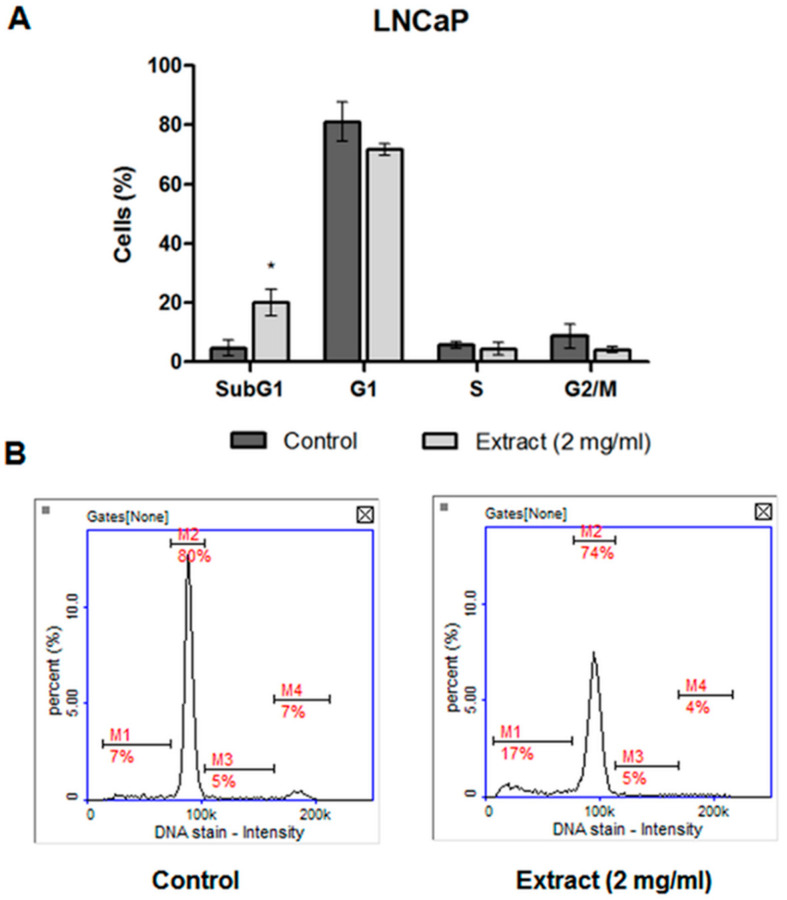
(**A**) Cell cycle analysis in LNCaP cells by image cytometry. The LNCaP cells were treated with 2 mg/mL of CA extract or DMSO as a vehicle in control cultures for 48 h. Data were presented as mean ± SD obtained from three independent experiments. * *p* < 0.05 vs. control. (**B**) Representative histograms (M1—subG1, M2—G1, M3—S, M4—G2/M phase).

The analysis of the cell cycle by image cytometry revealed changes in the populations of the LNCaP cells treated with the extract, compared to the control cells. A statistically significant increase in the peak corresponding to the population of LNCaP cells in the sub-G1 phase was noted, with a simultaneous decrease in the peak corresponding to the cells in the G1 phase (Figure 6). The results obtained in the cell cycle analysis confirmed the occurrence of the cytotoxic effect indicated in the MTT test in the LNCaP prostate cancer cell line treated with the CA extract at the concentration of 2 mg/mL.

#### 2.4.4. Apoptosis Detection

**Figure 7:**
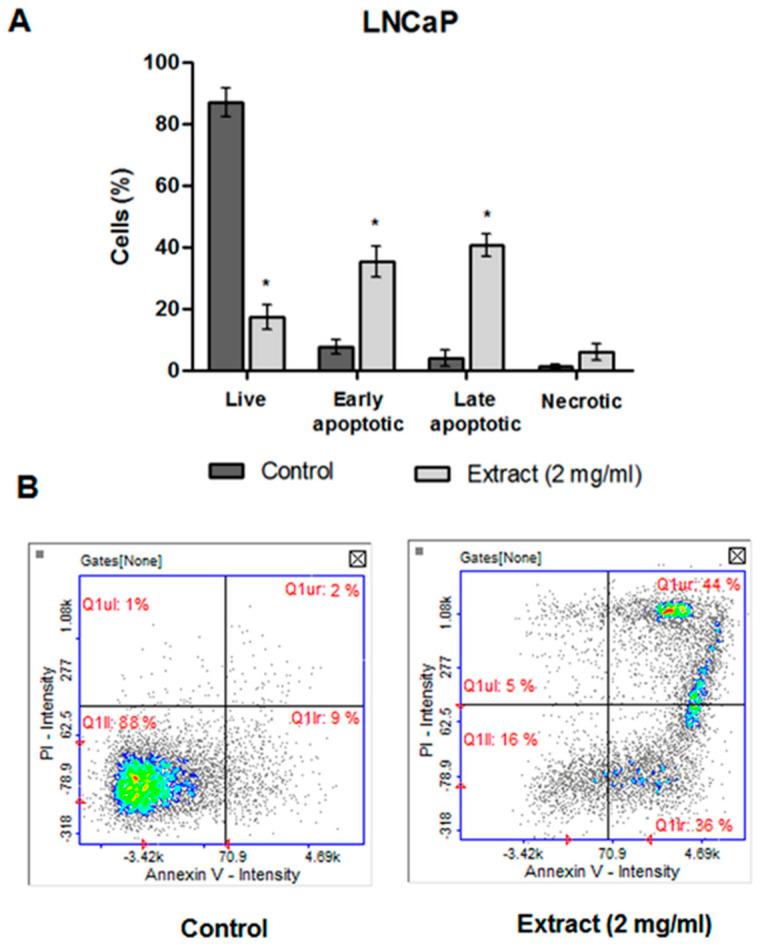
(**A**) Detection of cell apoptosis in LNCaP cells by image cytometry (Annexin V-FITC and propidium iodide staining). The LNCaP cells were treated with 2 mg/mL of CA extract or DMSO as a vehicle in control cultures for 48 h. Data were presented as mean ± SD obtained from three independent experiments. * *p* < 0.05 vs. control. (**B**) Representative histograms (Q1II—live, Q1Ir—early apoptotic, Q1ur—late apoptotic, and Q1uI—necrotic cells).

The cell death detected in the subG1 phase of the cell cycle was certainly apoptotic, according to the image cytometry analysis of apoptosis (Figure 7A). In the histogram, which represented the LNCaP cells treated with CA extract, the percentage of cells in the early and late stage of apoptosis (36%—early apoptotic phase; 44%—late apoptotic phase) were significantly increased in comparison to the control cells (Figure 7B).

#### 2.4.5. The Level of Cellular Thiols

**Figure 8:**
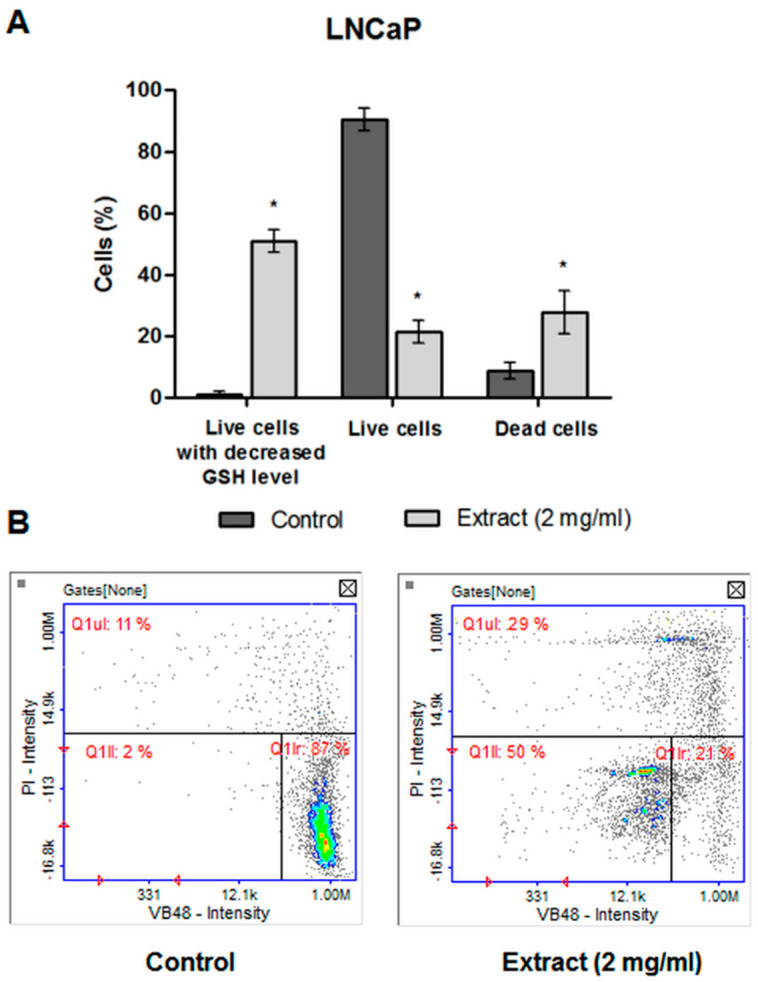
(**A**) Detection of the level of cellular thiols in LNCaP cells by image cytometry. The LNCaP cells were treated with 2 mg/mL of CA extract or DMSO as a vehicle in control cultures for 48 h. Data were presented as mean ± SD obtained from three independent experiments. * *p* < 0.05 vs. control. (**B**) Representative histograms (Q1II—PI negative cells with decreased GSH level, Q1Ir—healthy cells, Q1ur—dead cells).

The level of cellular thiols was performed to determine whether the tested CA extract causes disturbances in the level of intracellular glutathione (GSH) in LNCaP cells. GSH is a key component in the cellular antioxidant defense system, and disturbances in its level indicate a redox imbalance in the cell. The test showed that approximately 50% of the live LNCaP cells treated with the tested extract had a significantly decreased GSH level as compared to the control cells (Figure 8). This may indicate a redox imbalance caused by the tested extract, i.e., its pro-oxidative properties.

## 3. Discussion

Many of the over 85 *Crocus* species, including *C. alatavicus,* have been poorly or not yet studied in terms of their chemical composition and biological activity. The leaves and flowers of *Crocus* spp. can be regarded as an important source of several bioactive compounds, such as flavonoids, hydroxycinnamic acids and anthocyanins. The phytochemical profiling of *Crocus* spp. revealed the presence of 61 flavonoids and their glycosides [1]. Among them, kaempferol represents one of the most common aglycone flavonoid in the form of a glycoside [10].

The ethanolic extract from the aerial part of *C. alatavicus* growing wildly in southern Kazakhstan used in the present paper consisted mainly of leaves and flowers. The HPLC/ESI-QTOF-MS analysis revealed the presence of multiple kaempferol derivatives, which proves that the CA extract is a rich source of these compounds. Moreover, kaempferol 3-*O*-dihexoside (150.76 μg/mg of dry extract) and kaempferol 3-*O*-acyltetrahexoside (80.04 μg/mg of dry extract) were not only present in significant concentrations but also accounted for more than 70% of all the identified flavonoids and their derivatives. Some other flavonoids, such as derivatives of myricetin, quercetin and kaempferol, mainly rhamnosides, have been identified by HPLC in flowers of *C. alatavicus* from the Botanical Garden, University of Kopenhagen. As reported elsewhere, kaempferol glycosides constituted between 70 and 90% of the total contents of flavonoids in the flowers of the *Crocus* genus [2].

Satybaldiyeva et al. [8] identified nine phenolic acids in the ethanolic extract of the aerial part of *C. alatavicus* growing in Kazakhstan, namely gallic acid, 3,4-dihydroxybenzoic acid, 4-hydroxy-benzoic acid, chlorogenic acid, vanillic acid, caffeic acid, p-coumaric acid, ferulic acid, cinnamic acid. In the present paper, carboxyvanilic acid, as well as other acids, were detected in the CA extract, e.g., citric, malic and gluconic acids.

Polyphenols, including flavonoids, are widely distributed plant secondary metabolites with well-documented biological activity, mainly antibacterial, antifungal, antiviral and anticancer properties [11,12,13]. The total polyphenol content in the CA extract in the present study was 64.84 ± 0.88 mg GAE (gallic acid equivalent)/g (data not shown) and was comparable to the data presented by Satybaldiyeva et al. [8].

The ethanolic extract of *C. alatavicus* rich in kaempferol derivatives in the study presented in this paper was found to possess some antibacterial and antifungal activity, showing bactericidal and fungicidal effects. However, the obtained MIC values were higher than 1 mg/mL (10 mg/mL for yeasts and 20 mg/mL for bacteria). It should be noted that the breakpoint MIC of ≤ 1 mg/mL for plant extracts may indicate their noteworthy antimicrobial (antibacterial) activity, and suggests that further research is needed [14].

Some antiviral activity was observed in this paper at the *C. alatavicus* ethanolic extract concentration of 2 mg/mL, especially against HSV-1. This resulted in the inhibition of the virus-induced cytopathic effect and the reduction in virus infectious titer by 1.96 log, as well as the viral load by 0.85 log. This observation, together with the presence of high amounts of kaempferol derivatives in the CA extract, suggests that these compounds may be responsible for its antiherpetic activity. According to the literature data [15], several flavonoids, including kaempferol, showed a high inhibitory effect against HSV-1, measured by the virus-induced cytopathic effect and the plaque reduction assay. However, the antiherpetic activity of kaempferol derivatives was found to be variable, depending on the substituent. The most active against herpesviruses appeared to be kaempferol glucosides [16,17].

As reported elsewhere [8], the ethanolic extract from the aerial part of *C. alatavicus* growing in Kazakhstan was suggested to possess the potential anticancer activity based on data from the brine shrimp assay. In this paper, significant cytotoxic activity of the extract against the LNCaP cell line was found with an IC_50_ value of 1.95 mg/mL. The cell line used in the present study is derived from a metastatic lymph node lesion of human prostate cancer, which is androgen receptor (AR) positive and may be a useful model to study prostate cancer metastasis and to test experimental diagnostics and therapies for the treatment of advanced prostate cancer [18]. The LNCaP cell line treated with 2 mg/mL of the extract showed a noticeably reduced number of cells with a more spindle-shaped shape and longer cellular projections, a statistically significant increase in the peak corresponding to the population of cells in the sub-G1 phase of apoptotic characteristic and a significantly decreased GSH level, suggesting rather prooxidative properties of the extract. The anticancer activity of the extract studied may be due to the high amounts of kaempferol derivatives. As reported by Halimah et al. [19], kaempferol-3-*O*-rhamnoside dose-dependently inhibited prostate cancer cell proliferation.

It is worth noting that the spontaneous flora of Kazakhstan, consisting of many endemic and rare species [20,21], can be regarded as a rich source of several, not fully explored medicinal plants with novel potential applications [22,23,24,25,26,27,28,29]. Therefore, attempts have been made to introduce them for cultivation [30]. Recently, Allambergenova et al. [31] developed a procedure allowing for the cultivation of *C. alatavicus* from seeds according to the principles of good cultivation practice. Cultivation was carried out at the experimental plantation site of the pharmaceutical company “Fitoleum” (Almaty region in the village of Tole Bi; N 43°23′15′′ E 77°33′11.016′′). The ability to cultivate *C. alatavicus* creates novel possibilities to study and exploit this pharmacologically relevant endemic plant.

## 4. Materials and Methods

### 4.1. Plant Material Collection and Preparation

The collection of *C. alatavicus* raw materials was carried out in mid-March 2021 in the Almaty region at coordinates N 43.0106, E 79.1327, at an altitude of 1848 m above sea level. The aerial parts of the plant under study consisted of leaves and flowers. Since the *C. alatavicus* stem is underdeveloped, the leaves and flowers grow directly from the corm. The certificate of plant identification (N 01-08/105) was issued by specialists of the Institute of Botany and Phytointroduction of the Ministry of Ecology, Geology and Natural Resources of the Republic of Kazakhstan. The aerial parts of the *C. alatavicus* plant dried by the air-shade method were used for extraction.

### 4.2. Extraction Procedure

First, 10 g of crushed air-dry vegetable raw materials of *Crocus alatavicus* Regel et Semen (leaves and flowers) were placed in an ultrasonic extractor, then 100 mL of 96% ethyl alcohol was poured. Extraction was carried out under the influence of ultrasound with a frequency of 30 kHz at 25 °C, for 15 min, after which the extraction of biologically active substances was continued by maceration at the same temperature for 12 h. After that, the extract was filtered out, and the extraction process was repeated twice more under the conditions described above. The obtained extracts were combined and concentrated dry at a temperature of 40–50 °C and pressure of *p* = 190 Mbar. The resulting ethanol extract (1.72 g) was used for further studies.

### 4.3. Analysis of the CA Extract by RP-HPLC/PDA

The analysis of polyphenolic constituents by high-performance reverse-phase liquid chromatography combined with a photodiode-array detection (RP-HPLC/PDA) was described previously [22]. Briefly, the extract was purified using solid-phase extraction (SPE). A vacuum system (SPE-12G; J.T. Baker) connected to a vacuum pump (AGA-Labor, Warsaw, Poland) was used. An Agilent Technologies (Waldbronn, Germany) 1100 Series liquid chromatograph equipped with an autosampler and a PDA detector, set at 254 nm, 280 or 325 nm was applied. Chromatographic separations were carried out on a Zorbax Eclipse XDB C8 column (150 × 4.6 mm I.D., dp = 5 μm). The following reference substances were used in the study: kaempferol and rutoside (Sigma-Aldrich, St. Louis, MO, USA), astragalin and nicotiflorin (ChromadDex, Los Angeles, CA, USA). The amount of kaempferol and quercetin derivatives were calculated adequately on the amount of kaempferol or quercetin. Calibration curves were established for the four standard substances using six different concentrations (0.1, 0.075, 0.05, 0.025, 0.0125 mg/mL). The following equations were obtained: kaempferol y = 22.544x + 889.22; R^2^ = 0.9954. quercetin y = 8283.4x + 394.92; R^2^ = 0.9979. rutoside y = 49.694x − 31.317; R^2^ = 0.9989. astragalin y = 10,581x + 42.299; R^2^ = 0.9964. nicotiflorin y = 8595.6x + 14.53; R^2^ = 0.9966. Analyses were performed during the three consecutive days. The repeatability of the peak areas was determined by the evaluation of SD. The mean values ± SD were presented.

### 4.4. Analysis of the CA Extract by HPLC/ESI-QTOF-MS

The CA extract was analyzed qualitatively by a HPLC/ESI-QTOF-MS system in negative ion mode with the use of a 6530B Accurate-mass-QTOF-MS (Agilent Technologies, Inc., Santa Clara, CA, USA) mass spectrometer with an ESI-Jet Stream ion source by the procedure described previously [22]. The Agilent 1260 infinity chromatograph was equipped with a DAD detector, autosampler, binary gradient pump and column oven. The column used as the stationary phase was the Gemini^®^ 3 µm NX-C18 110 Å, HPLC Column 100 × 2 mm. A gradient of solvents: water with 0.1% formic acid (solvent A) and acetonitrile with 0.1% formic acid (solvent B) were used as the mobile phases. The following gradient procedure was adopted: 0–45 min, 0–60% of B; 45–46 min, 60–90% B; 46–50 min, 90% (B); the post time was 10 min. The total time of analysis was 60 min, with a stable flow rate at 0.200 mL/min. The injection volume for extracts was 10 μL. ESI-QTOF-MS analysis was performed according to the following parameters of the ion source: dual spray jet stream ESI, positive and negative ion mode, gas (N_2_) flow rate: 12 L/min; nebulizer pressure: 35 psig; vaporizer temperature: 300 °C; *m*/*z* range 100–1000 mass units, with acquisition Mode Auto MS/MS, collision induced dissociation (CID): 10 and 40 V with MS scan rate 1 spectrum per s, 2 spectra per cycle; skimmer: 65 V; fragmentor: 140 V and octopole RF peak: 750 V. The identification of compounds was based on Metlin (https://metlin.scripps.ed (accessed on 23 May 2022).

### 4.5. Determination of Antibacterial and Antifungal Activity

The assay of antibacterial and antifungal activity of the CA extract was performed by broth microdilution method, allowing for the determination of MIC as well as MBC or MFC, according to the European Committee on Antimicrobial Susceptibility Testing [32]. Mueller–Hinton broth (for bacteria) and Mueller–Hinton broth with 2% glucose (for yeasts) were used. The tested microorganisms were the reference strains from the American Type Culture Collection (ATCC), including *E. coli* ATCC 25922 (Gram-negative bacterium), *S. aureus* ATCC 29213, *S. aureus* ATCC BAA-1707, *S. epidermidis* ATCC 12228, *B. cereus* ATCC 10876, *C. acnes* ATCC 11827 (Gram-positive bacteria), and yeasts *C. albicans* ATCC 10231 and *C. glabrata* ATCC 90030. Among all the reference strains of microorganisms used in this study, only *S. aureus* ATCC BAA-1707 that belongs to methicillin-resistant *S. aureus* (MRSA) is characterized by an acquired type of resistance to antibiotics (β-lactams). The antimicrobial assays were performed as described in detail previously [22,33]. Vancomycin (0.03–10 mg/mL), ciprofloxacin (0.007–10 mg/mL) and fluconazole (0.03–10 mg/mL) were included as reference antimicrobial substances active against Gram-positive bacteria, Gram-negative bacteria or yeasts. Each experiment was repeated in triplicate. Of the three MIC and MBC or MFC values, the most common representative value, i.e., the mode, was presented.

### 4.6. Determination of Antiviral Activity

The cytotoxicity of the CA extract was tested in vitro towards normal VERO (ATCC, No. CCL81) cells using the MTT-based protocol. The evaluation of the antiviral activity of the CA extract was carried out against HSV-1 (ATCC, No. VR-260) and CVB3 (ATCC No. VR-30) propagated in the VERO cell line. The antiviral assays included the influence of the tested extract on the formation of HSV-1 or CVB3-induced cytopathic effect and end-point virus titration for infectious titer.

The Dulbecco Modified Eagle Medium (DMEM, Corning, Tewksbury, MA, USA) was used for VERO cell culturing, whereas cancer cell lines were grown in Modified Eagle Medium (MEM, Corning). All the cell media were supplemented with antibiotics (Penicillin-Streptomycin Solution, Corning) and fetal bovine serum (FBS, Capricorn). The trypsin and phosphate-buffered saline (PBS) were purchased from Corning, whereas DMSO and MTT were from Sigma (Sigma-Aldrich, St. Louis, MO, USA). The cells were maintained in a 5% CO_2_ atmosphere at 37 °C (CO_2_ incubator, Panasonic Healthcare Co., Tokyo, Japan).

The CA extract was dissolved in DMSO (200 mg/mL) to obtain a stock solution. The stock solution was stored frozen until used in experiments.

#### 4.6.1. Cytotoxicity Assessment

Cytotoxicity was tested using the MTT based assay. Briefly, the cells were passaged into 96-well plates, and after overnight incubation, semiconfluent monolayers were treated with serial dilutions (4–0.0039 mg/mL) of the tested extract for 48 or 72 h. Afterwards, the medium was removed, the cells were washed with PBS, and 0.1 mL per well of 10% MTT solution (5 mg/mL) in culture media was added and the plates were incubated for the next 3 h. Subsequently, 0.1 mL per well of SDS/DMF/PBS (14% SDS, 36% DMF, 50% PBS) solution was added to dissolve the formazan product and the plates were incubated overnight. Finally, the absorbance at 540 and 620 nm was measured with a Synergy H1 Multi-Mode Microplate Reader (BioTek Instruments, Inc. Winooski, VT, USA) running Gen5 software (ver. 3.09.07; BioTek Instruments, Inc.). Data analysis was performed using GraphPad Prism (v7.0.4; San Diego, CA, USA) to calculate CC_50_ values from the dose–response curves (non-linear regression, normalized response), based on the comparison of absorbance recorded for the extract-treated and control cells.

#### 4.6.2. Antiviral Assay

The VERO cells were passaged into 48-well plates and after overnight incubation treated with HSV-1 or CVB3 in 100-fold CCID_50_/mL (50% cell culture infectious dose) and incubated for 1 h, leaving uninfected wells as the cell control. Afterwards, the cells were washed with PBS, and treated with non-toxic concentrations of the extracts; the highest concentration did not decrease the VERO cell viability by more than 10%. The non-infected VERO cells (cell control) and non-treated infected cells (virus control) wells were supplemented with media containing 2% FBS. The plates were further incubated until a typical CPE was observed in the virus control. Routine observation of CPE was carried out using an inverted microscope (CKX41, Olympus Corporation, Tokyo, Japan) equipped with a camera (Moticam 3+, Motic, Hong Kong) and the effect of the extracts on CPE formation in comparison with the virus control was documented (Motic Images Plus 2.0, Motic). Subsequently, the plates were thrice frozen (−72 °C) and thawed, and the samples were collected and kept frozen at −72 °C until virus titration assay and viral DNA isolation. The selectivity index (SI) was calculated for the samples that inhibited the formation of virus-induced CPE according to the formula SI = CC_50_/MAC; CC_50_—concentration of sample decreasing the viability of VERO cells by 50%; mac—minimal concentration of sample visibly inhibiting the formation of virus-induced CPE.

#### 4.6.3. End-Point Dilution Assay for HSV-1 Infectious Titre

The monolayers of VERO cells in 96-well plates were treated with 10-fold dilutions (10^−1^–10^−12^) of the samples collected from antiviral assays and incubated for 72 h. The plates were observed daily using an inverted microscope. Afterwards, the medium was removed and the MTT method described above was used to assess the viability of cells treated with different dilutions of samples, and the collected data were used to estimate the CCID_50_/_mL_ values using GraphPad Prism software. In order to evaluate the degree of antiviral activity, the difference of viral load (Δlog) in the virus control (logCCID_50_VC) and extract-treated samples (logCCID_50_E) was calculated using the following formula: Δlog = logCCID_50_VC—logCCID_50_E. The Δlog values were calculated for every end-point dilution assay and presented as mean Δlog. Significant antiviral activity can be reported for the samples that inhibited the virus titer by at least 3 log.

#### 4.6.4. Real-Time PCR for HSV-1 Viral Load

The DNA isolation was carried out using a commercially available kit (QIAamp DNA Mini Kit, QIAGEN GmbH, Hilden, Germany) following the manufacturer’s instructions. The real-time PCR amplification was performed using TB Green Advantage qPCR Premixes (Takara Bio, Mountain View, CA, USA) and primers (UL54F—5′ CGCCAAGAAAATTTCATCGAG 3′, UL54R—5′ ACATCTTGCACCACGCCAG 3′) on the Rotor-Gene Q (QIAGEN) thermal cycler. The amplification cycle parameters were as follows: initial denaturation (95 °C, 20 s); cycling (45 repeats: denaturation (95 °C, 5 s), annealing/synthesis (60 °C, 30 s), fluorescence acquisition (Green); melting curve analysis (60–95 °C). The quantitative analysis was carried out using a calibration curve comprised of tenfold dilutions of HSV-1 DNA isolate, which were previously quantitatively analyzed using the IVD certified GeneProof Herpes Simplex Virus (HSV-1/2) PCR Kit (GeneProof a.s., Czech Republic).

### 4.7. Anticancer Activity

#### 4.7.1. Cell Culturing and Treatment

The studies were performed on three prostate cancer cell lines (PC-3, DU145 and LNCaP) (ATCC, Manassas, VA, USA), and the skin fibroblast cell line (BJ) (ATCC, Manassas, VA, USA) as a normal cell line. BJ cells were used to test whether the obtained CA extract was toxic at the tested concentrations. PC-3 cells were cultured in Kaighn’s Modification of Ham’s F-12 Medium (F12-K) (ATCC, Manassas, VA, USA), LNCaP cells in RPMI-1640 Medium (ATCC, USA) and both DU145 cells and BJ cells in Eagle’s Minimum Essential Medium (EMEM) (ATCC, Manassas, VA, USA). For cell culturing, the media were supplemented with 10% fetal bovine serum (Corning, Tewksbury, MA, USA) and were incubated at 37 °C with 5% CO_2_ air. The cells were seeded into plates at a concentration of 1.5 × 10^5^ cells/mL, and when 70–80% of cell confluence was achieved, the extract was added. The cells were incubated for 48 h with CA extract in a wide range of concentrations (2–0.1 mg/mL) or DMSO as a vehicle in the control cultures (maximal DMSO concentration <0.5%). The cytotoxicity effect of the CA extract was evaluated with the use of the MTT assay, as described above in point 4.6.1.

#### 4.7.2. Assessment of Cell Morphology and Cell Cycle Analysis

To evaluate the effect of the CA extract on the morphology of the cells, a Nikon Eclipse TI optics inverted microscope, a phase-contrast microscope as well as NIS-Elements Imaging Software (Nikon Instruments Inc., Melville, NY, USA) were used.

The cell cycle analysis was carried out using NucleoCounter^®^ NC-3000™ (ChemoMetec, Allerod, Denmark), following a 2-step Cell Cycle Assay protocol (ChemoMetec, Allerod, Denmark). LNCaP cells were seeded into 6-well plates and treated with CA extract in the concentration of 2 mg/mL. After 48 h of incubation, the culture medium was removed, and the cells were detached from the plate using trypsin-EDTA solution (Corning, Tewksbury, MA, USA). Then, the cells were washed once with PBS, suspended in 100 μL of lysis buffer (Solution 10), supplemented with 10 μg/mL of DAPI (4′,6-diamidine-2′-phenylindole dihydrochloride) and incubated in the dark for 5 min at 37 °C. Next, 100 μL of stabilization buffer (Solution 11) was added, and 10 μL of the cell suspension was loaded into NC-Slide and analyzed in NucleoCounter NC-3000. The experiment was conducted three times with measurements in triplicate.

#### 4.7.3. Detection of Apoptosis

Apoptosis detection assay was performed using NucleoCounter^®^ NC-3000™ (ChemoMetec, Allerod, Denmark), following the Annexin V Apoptosis Assay (ChemoMetec, Allerod, Denmark). LNCaP cells were seeded into 6-well plates. After 48 h of incubation with the CA extract in the concentration of 2 mg/mL, the culture medium was removed, the cells were detached from the plate using trypsin-EDTA solution and washed once with PBS. Then, the cell pellet was carefully resuspended in 100 μL of Annexin V binding buffer enriched with 2 μL of Annexin V-CF488A conjugate and 4 μL of Hoechst 33342 (10 μg/mL) and incubated for 15 min at 37 °C. After incubation, the cells were centrifuged at 400 × g for 5 min. Next, the supernatant was removed and 100 μL of Annexin V binding buffer supplemented with 10 μg/mL of PI was added to the cells. The obtained cell suspension was applied into NC-Slide at a volume of 30 μL and analyzed in NucleoCounter NC-3000. The experiment was conducted three times with measurement in triplicate.

#### 4.7.4. The Research of Oxidative Stress by the Level of Cellular Thiols

The level of cellular thiols in LNCaP cells was carried out using theNC-3000 Vitality Assay (Chemometec, Allerod, Denmark). The principle of the method is based on the reaction of the Vita Bright-48 dye with thiols and the formation of a fluorescent product. Other dyes used in this staining were as follows acridine orange (AO; stains only dead cells) and propidium iodide (PI; stains all nucleated cells).

LNCaP cells were seeded into 6-well plates. After 48 h of incubation with the CA extract in the concentration of 2 mg/mL, the culture medium was removed, the cells were detached from the plate using trypsin-EDTA solution and washed once with PBS. Then, the LNCaP cells were suspended in Solution 5 (solution containing all three dyes used in the assay) at a 19/1 ratio. A total of 10 µL of obtained cell suspension was applied into NC-Slide and analyzed in NucleoCounter NC-3000. The experiment was conducted three times with measurements in triplicate.

#### 4.7.5. Statistical Analysis

The obtained data were given as mean ± SD and analyzed by Statistica 13 software (StatSoft, Krakow, Poland). The statistical significance was evaluated by Student’s t-test; * *p* ≤ 0.05 was considered statistically significant.

## 5. Conclusions

*C. alatavicus*, a representative of Kazakhstan’s spontaneous and endemic flora, can be regarded as a valuable source of biologically active compounds, especially kaempferol derivatives. The comprehensive analysis of *C. alatavicus* extract presented in this paper revealed its noticeable antiviral effect towards HHV-1, as well as significant anticancer activity against metastatic prostate cancer. It is important to highlight that the ability to cultivate *C. alatavicus* from seeds creates new possibilities to screen this endemic plant for a wide range of biological properties and to use it on a large-scale level in the pharmaceutical industry.

## 6. Patents

The method for cultivating Alatau saffron by seeds includes the patent for utility model no. 6737, Republic of Kazakhstan, IPC A01H 5/00 (2006.01), A01H 5/10 (2006.01). This process involved Allambergenova Zoya Baqbergenqyzy, Sakipova Zuriyadda Bektemirovna, Aliev Nysanali Uzhetbaevich, Sermukhamedova Olga Vladimirovna, Otradnykh Irina Gennadevna, Sedina Irina Anatolevna. The applicant and patent holder are as follows: Asfendiyarov Kazakh National Medical University, 2021/0774.2 and the application submission date was 9 August 2021.

## Figures and Tables

**Table 1:** Chromatographic data of compounds identified by HPLC/ESI-QTOF-MS in CA extract.

No	Compound	Retention Time (min)	Formula	Molecular Ion [M − H]^−^	Fragmentation Ions
1.	Gluconic acid	1.917	C_6_H_12_O_7_	195.0512	177.0421; 129.0195; 99.0094; 75.0095
2.	Malic acid	2.041	C_4_H_6_O_5_	133.0145	115.0043; 71.0148
3.	2-Deoxy-2,3-dehydro-n-acetyl- neuraminic acid	2.273	C_11_H_17_NO_8_	290.0891	200.0561; 170.0454; 128.0352
4.	Citric acid	2.459	C₆H₈O₇	191.0200	129.0191; 111.0091; 87.0095
5.	DH-Crocusatin F	4.994	C_10_H_16_O_4_	199.0973	155.1072; 137.0969; 125.0977; 111.0821
6.	Crocusatin F	5.864	C_10_H_14_O_4_	197.0840	153.0939; 137.0985; 125.0987; 111.0831
7.	Kaempferol 7-*O*-tetrahexoside	12.109	C_39_H_50_O_26_	933.2560	771.1928; 609.1475; 446.0885; 284.0339
8.	Kaempferol 3-*O*-acyltetrahexoside	12.799	C_43_H_54_O_26_	973.2846	771.2038; 609.1481; 446.0870; 284.0340
9.	2,4,4-Trimethyl-3-formyl-6-hydroxy-2,5-cyclohexadien-1-one	13.029	C_10_H_12_O_3_	179.0722	135.0801; 120.0578; 109.0305
10.	Kaempferol 7-*O*-dihexosidee	16.973	C_27_H_30_O_16_	609.1454	447.0840; 285.0351
11.	Kaempferol 7-*O*-trihexoside	20.847	C_33_H_40_O_21_	771.1996	447.0847; 285.0373
12.	Quercetin 3-*O*-dihexoside	20.997	C_27_H_30_O_17_	625.1438	300.0292; 271.0247
13.	Kaempferol 3,7-rutinoside, dihexoside	21.331	C_39_H_50_O_25_	917.2568	771.1673; 755.1738; 609.1419; 593.1419; 285.0337; 284.0281
14.	Kaempferol 3-*O*-dihexoside	22.335	C_27_H_30_O_16_	609.1470	284.0335; 255.0297
15.	Rutoside	23.172	C_27_H_30_O_16_	609.1486	300.0269; 284.0319; 271.0245
16.	Kaempferol 7-*O*-rutinoside	24.677	C_27_H_30_O_15_	593.1540	285.0372
17.	Astragalin	25.514	C_21_H_20_O_11_	447.0944	284.0322; 255.0299; 227.0343; 151.0036
18.	Nicotiflorin	26.267	C_27_H_30_O_15_	593.1479	284.0311; 255.0292
19.	Carboxyvanilic acid	27.609	C_9_H_8_O_6_	211.0240	167.0353; 123.0454; 108.0227
20.	Kaempferol	34.420	C_15_H_10_O_6_	285.0425	229.0476; 185.0605; 135.0077; 109.0288
21.	Endocrocin	35.119	C_16_H_10_O_7_	313.0379	241.0506; 225.0565; 213.0555; 201.0576; 197.0604
22.	Acacetin	42.691	C_16_H_12_O_5_	283.0614	268.0382; 240.0433; 212.0482

**Table 2:** Flavonoid content in CA extract.

Compound	Retention Time (min)	Content (μg/mg Dry Extract)		
Kaempferol 3-*O*-tetrahexoside	2.11	14.14	0.09 ^a^	0.6 ^b^
Kaempferol 3-*O*-acyltetrahexoside	2.47	80.04	0.51 ^a^	0.6 ^b^
Kaempferol 7-*O*-trihexoside	9.10	9.32	0.22 ^a^	2.4 ^b^
Quercetin 3-*O*-dihexoside	9.40	6.89	0.07 ^a^	1.0 ^b^
Kaempferol 3,7–rutinoside, dihexoside	9.54	10.51	0.09 ^a^	0.9 ^b^
Kaempferol 3-*O*-dihexoside	12.77	150.76	0.16 ^a^	0.1 ^b^
Rutoside	13.50	5.12	0.04 ^a^	0.7 ^b^
Kaempferol 7-*O*-rutinoside	13.97	4.71	0.10 ^a^	2.2 ^b^
Astragalin	14.47	26.87	0.25 ^a^	0.9 ^b^
Nicotiflorin	15.83	30.83	0.26 ^a^	0.9 ^b^
TOTAL (μg/mg dry extract)		339.19		

^a^ SD—standard deviation (*n* = 3); ^b^ RSD—relative standard deviation.

**Table 3:** The activity of CA extract towards bacterial and fungal reference strains.

**Bacteria**	**MIC ^1^ (mg/mL)**	**MBC ^2^ (mg/mL)**	**MBC/MIC Ratio**
*Escherichia coli* ATCC 25922	20	>20	Nd ^4^
*Staphylococcus aureus* ATCC 25923	20	20	1
*Staphylococcus aureus* ATCC BAA-1707	20	20	1
*Staphylococcus epidermidis* ATCC 12228	20	>20	Nd
*Bacillus cereus* ATCC 10876	20	20	1
*Cutibacterium acnes* ATCC 11827	20	20	1
**Fungi (Yeasts)**	**MIC (mg/mL)**	**MFC ^3^ (mg/mL)**	**MFC/MIC Ratio**
*Candida albicans* ATCC 10231	10	10	1
*Candida glabrata* ATCC 90030	10	20	2

^1^ Minimal inhibitory concentration, ^2^ minimal bactericidal concentration, ^3^ minimal fungicidal concentration, ^4^ not determined.

**Table 4:** Reduction in infectious titer and viral load by the CA extract.

Concentration (mg/mL)	Reduction in Infectious Titer (Δlog) ^1^		Reduction in Viral Load (Δlog’) ^2^	
	CVB3	HSV-1	CVB3	HSV-1
2	1.14 ± 0.38	1.96 ± 0.28	Nd ^3^	0.85 ± 0.10
1	0.55 ± 0.31	0.90 ± 0.06	nd	0.24 ± 0.07

^1^ Δlog (mean ± SD)—mean was calculated from titration assays of samples from different antiviral assays; Δlog = logCCID_50_VC—logCCID_50_E; VC—virus control; E—tested extract, Δlog of at least 3 is regarded as significant; ^2^ Δlog’ (mean ± SD)—mean was calculated from samples from different antiviral assays; Δlog’ = log(copies/mL)VC—log(copies/mL)E; VC—virus control; E—tested extract; ^3^ not determined.

## Sample Availability

SSamples of the compounds are not available from the authors.
